# Sol-Gel Synthesis of Non-Silica Monolithic Materials

**DOI:** 10.3390/ma3042815

**Published:** 2010-04-21

**Authors:** Bartłomiej Gaweł, Kamila Gaweł, Gisle Øye

**Affiliations:** Ugelstad Laboratory, Department of Chemical Engineering, Norwegian University of Science and Technology (NTNU), N-7491 Trondheim, Norway; E-Mails: bartlomiej.gawel@chemeng.ntnu.no (B.G.); kamila.gawel@ntnu.no (K.G.)

**Keywords:** monoliths, hierarchical porosity, sol-gel

## Abstract

Monolithic materials have become very popular because of various applications, especially within chromatography and catalysis. Large surface areas and multimodal porosities are great advantages for these applications. New sol-gel preparation methods utilizing phase separation or nanocasting have opened the possibility for preparing materials of other oxides than silica. In this review, we present different synthesis methods for inorganic, non-silica monolithic materials. Some examples of application of the materials are also included.

## 1. Introduction

According to the IUPAC definition: “A monolith is a shaped, fabricated, intractable article with a homogeneous microstructure that does not exhibit any structural components distinguishable by optical microscopy” [1]. A compact monolithic structure is very often an advantage, or even a requirement, for many applications. For example, porous monoliths used in flow through catalytic or separation systems give lower backpressure, higher permeability and better performance compared to packed columns [2]. Inorganic multiporous monoliths based on silicon oxide are the most common. Applications include solid phase micro extraction [3], separation [4] and catalysis [5]. Porous monoliths based on alumina, titania, zirconia or carbon can broaden the application areas further. Such monoliths can also exhibit large surface areas and pore volumes, and the presence of macropores ensures high permeability. These advantages, accompanied with mechanical, chemical and thermal stability, and special surface properties, can lead to novel applications of the materials. Some examples are given below.

Monolithic TiO_2_ rod columns were used to separate a mixture of adenosine triphosphate, adenosine diphosphate and adenosine monophosphate [6]. The separation efficiency for the columns was due to their ability to bind phosphate groups. The binding strength (and the retention time) increased with increasing number of phosphate groups. The number of theoretical plates increased when the macropore size decreased, but was accompanied by higher backpressures. However, in contrast to standard particle-packed column with the same dimensions, monolithic columns exhibited lower backpressure and higher efficiency. Silica columns are inactive in separation of these kinds of compounds due to the lack of interaction between silica and phosphate groups. Monolithic titania columns were also used by Randon *et al.* [7] for separation of naphthalene, caffeine, 7-(β-hydroxyethyl)theophylline and theophylline in hydrophilic interaction liquid chromatography (HILIC). The same technique was applied for separation of amines (mixture consisting of naphthalene, orthotoluidine and aniline) and an alkoxybenzene mixture (mixture consisting of thiourea, toluene, ethylbenzene, propylbenzene), using zirconia monoliths as the stationary phase [8].

Alumina monoliths modified with platinum and potassium were applied as catalysts for preferential CO oxidation in hydrogen-rich stream and showed spurious support properties compared to macroporous honeycomb structured and microporous reactors [9]. Hierarchically porous carbon monolith (HPCM) has been reported to be effective supports for supercapacitive materials like aniline or LiFePO_4_ [10,11]. The monolithic shape can also be an advantage in luminescent [12] or superionic conductivity application [13]. Introduction of Co atoms into ZrO_2_ monolithic matrices and Brilliant Blue dye molecules or Pr atoms into titania matrices, induced luminescence or enlarged the absorption spectra range [14,15,16] in the materials. Strongly scattering TiO_2_ is a promising random laser material within photonics: the laser action can be obtained by the interference of multiply scattered light from organic dyes incorporated in the pores [17].

Monoliths can be fabricated by cold or hot pressing of polymeric materials, or by reactive processing techniques such as reaction injection molding, crosslinking, sol-gel processing and sintering [1]. Particularly, the sol-gel process has become a very popular fabrication method for inorganic monoliths, as materials with high specific surface areas, multimodal ordered porosity and high homogeneity can be prepared. The methods for preparing silica and non-silica monoliths are principally the same. However, development of the latter was limited for many years, due to high hydrolysis rates of the precursors. Subsequent developments of phase separation and replica techniques, in addition to using hydrolysis controlling additives, made it possible to overcome this. The number of publications within this field has flourished during the last few years, and the focus of the present paper is to review recent progress within sol-gel processing of inorganic, non-silica, monolithic materials.

## 2. Synthesis of Monolithic Materials

First a general overview of different synthetic approaches making use of the sol-gel technique is given. It comprises (1) phase separation approaches, (2) templating approaches and (3) replication of monolithic matrices.

### 2.1. The Sol-gel method

The sol–gel method is an important technique for processing of metal oxides and the technology has been known for two centuries. Since the first reports on preparation of inorganic monoliths in the early 1970s, the interest for the method has increased tremendously [4]. The process can be divided into the following general stages:

a) hydrolysis of precursors-sol formation
b) polycondensation of hydrolyzed precursors-gelation
c) aging
d) drying
e) calcination

Sols can be prepared from both organic and inorganic compounds. Hydrolysis and polycondensation reactions usually occur simultaneously and the reaction rates depend on the type of precursor as well as reaction conditions like pH, temperature and ionic strength. Hydrolysis of alkoxy precursors (≡M-OR_n_, where M is the metal atom and R is the alkyl group) occur due to nucleophilic substitution of alkoxy groups by water. The mechanism involves nucleophilic addition followed by proton transfer (see equation below). The hydrolyzed precursors (≡M-OH) can react either with alkoxy precursors (alcoxolation) or with other hydrolyzed precursors (oxolation). In both cases the result is polycondensation [18,19].

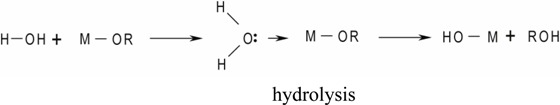
(1)

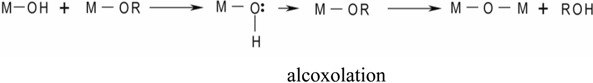
(2)

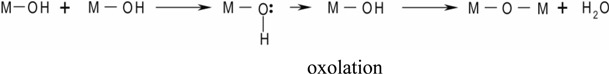
(3)

Protonation of alkoxide ligands occurs easily in the presence of acids. This can influence the polycondensation by formation of partially hydrolyzed inorganic polymers (the term inorganic polymers and polymerization will in this paper be used to describe the inorganic networks and polycondensation/gelation process, respectively). The condensation is then preferentially directed towards the chain ends, resulting in more linear (less branched) inorganic polymers [18].

The polycondensation, which macroscopically is seen as gelation, results in 3D rigid networks. The gel aging involves polymerization, syneresis and coarsening. Unreacted MOH and MOR groups condense during aging, resulting in increased connectivity and strength of the gel network. This can cause shrinkage of the gel network and results in syneresis (expulsion of liquid from the pores). Porosity differences in the material can also cause dissolution-precipitation processes to take place, where small particles disappear and small pores are filled (coarsening). Consequently the interfacial is decreased and the average pore size is increased [19].

The drying stage is often the crucial one during preparation of monoliths. Drying by simple evaporation results in xerogels. If the materials are dried using supercritical CO_2_ (scCO_2_), they are called aerogels. Recently ionic liquids have also been used during drying and calcination procedures, and the materials are then called ionogels [20].

When liquid evaporates from the porous gels, the pore walls are subjected to a stress equal to the capillary pressure. The stress can cause gel shrinkage and collapse of the pore walls. Moreover, the material can be exposed to pressure gradients between the wet and dry parts of the pores. This can also cause cracking of the monolithic structure. The capillary pressure depends on the properties of the material and the solvent, and is expressed with the Young-Laplace equation [19].

(4) $$P=\frac{2\gamma_{LV}\text{cos }\theta }{r}$$

where P is the capillary pressure, γ_LV_ is the liquid-vapor interfacial tension, θ is the contact angle and r is the pore radius.

The capillary pressure can be reduced in two ways: 1) decrease of the interfacial tension between liquid and vapor, 2) increase of the pore radius. Consequently, a common method used to avoid cracking of monolithic structures is to reduce the capillary pressure by exchange with or addition of a liquid with low surface tension (e.g. alcohols or DMF) [21,22]. Immersion of porous materials in glycerol after gelation have also been used to reduce cracking during standard drying [23] and freeze drying [24]. Supercritical drying is another example of the same approach. In the first step the water is exchanged by organic solvent and next by supercritical CO_2_. In supercritical CO_2_ the liquid-vapor interface and the capillary pressure are absent. Moreover, the low viscosity and high diffusivity of scCO_2_ make it easy to remove without collapsing the solid matrix [25]. Other solvents can also be used for supercritical drying [26,27], but the process becomes more complicated due to the higher temperature and pressure conditions required to reach the supercritical state. Introduction of macroporosity can also be used to retain the monolithic structure during drying [23]. This can be obtained by the phase separation or templating methods described in section 2.2 and section 2.3, respectively.

During calcination, all organic compounds are removed from the inorganic monolith by heating. Calcination often results in more mechanically stable materials, but sintering can cause the density of the materials to increase and the pore volume and surface area to decrease. The calcination stage is not always necessary, and in many cases organic compounds can be removed by washing or extraction.

### 2.2. Phase separation. Template-free synthesis

Preparation of monoliths with well defined porous structures can be obtained by combining gelation and phase separation. The phase separation is induced by the presence of a porogen during a sol-gel process. In most cases water soluble amphiphilic polymers are used as porogens. The molecular mechanism is believed to be as follows: During the gelation, the porogen is partially adsorbed onto the inorganic network, where the hydrophilic parts of the amphiphilic copolymer are involved in the adsorption. This reduces the number of effective hydrogen-bonding sites in the polymer molecule and results in a more hydrophobic environment around the binding sites. Due to increased hydrophobicity the solubility of the adsorbed polymer is lowered and phase separation is induced. [28] In other words, the porogen separates more easily together with adsorbed inorganic oligomers. The solubility in the system depends on the temperature as well as the molecular weight of both the porogen and the growing inorganic polymer. When the parameters are appropriately chosen, the phase separation can be “frozen” into inorganic gel networks. The concept “chemical cooling” is frequently used to describe this phenomenon [28].

Mixing, in the above described system, can be described by the Flory–Huggins equation:

(5) $$\text{Δ}G\propto RT\left(\frac{\phi_{1}}{P_{1}}\text{ln}\phi_{1}+\frac{\phi_{2}}{P_{2}}\text{ln}\phi_{2}+\chi_{12}\phi_{1}\phi_{2}\right)$$

where ΔG is the change of Gibbs free energy of mixing, $\phi_{i}$ and Pi (i = 1,2) denote the volume ratio and the degree of polymerization of each component respectively, and $\chi_{12}$ is a parameter describing the interaction between the components.

For polymers of high molecular weight, the entropic contribution is very small (the two first terms in Equation 5) and the miscibility or immiscibility of the system mainly depends on the value of the enthalpy of mixing (third term in Equation 5.). From a thermodynamic point of view, the change in Gibbs free energy of mixing increases during condensation of inorganic components. When ΔG is negative, the mixing is a spontaneous process [29]. Nevertheless a system with two mixed phases may be more stable than a homogeneous system. Then phase separation may occur. The phase separation may involve two different mechanisms (nucleation and spinodal decomposition) depending on the region in the phase diagram where it was induced.

**Figure 1:**
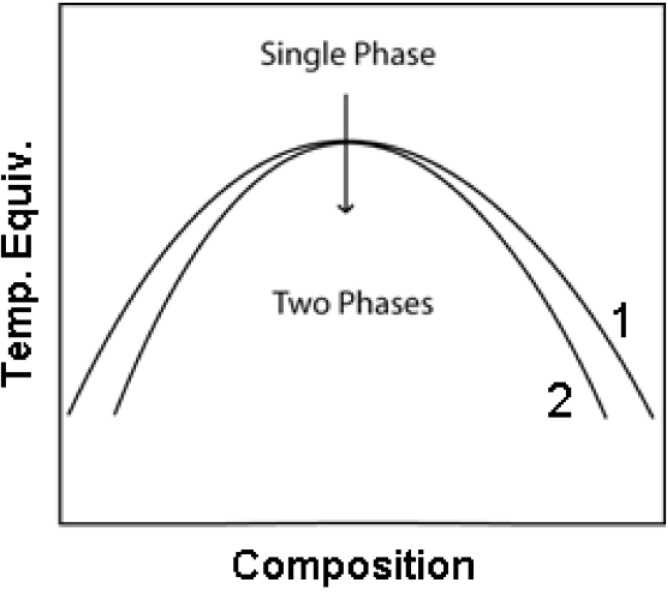
Phase separation diagram (1 and 2 denote binodal and spinodal lines, respectively).

Figure 1 shows a phase separation diagram, in which curve 1 shows where the first derivative of ΔG becomes 0 (binodal line) and curve 2 shows where the second derivative of ΔG becomes 0 (spinodal line). When phase separation is induced in the unstable region of the phase diagram (where the spinodal and binodal lines coincide), a process called spinodal decomposition occurs. During this process small fluctuations in the composition of the whole system occur. Their amplitude grows exponentially with time and the contrast between the phase domains arises. For a substantial period of time, the bicontinuous structure grows and remains stable. However, when the phase separation starts in the region between spinodal and binodal lines, the nucleation mechanism dominates and an inhomogeneous domain structure is formed.

**Figure 2:**
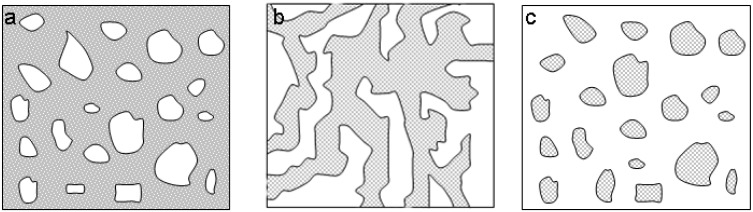
Different types of morphology which occur during coarsening of various starting compositions.

When the volume fractions of interconnected phase domains are comparable, a bicontinuous, spongelike structure forms (Figure 2b). This structure is characterized by continuous mutually conjugated domains with hyperbolic interfaces [29]. Figure 2 illustrates the structures that can be obtained by different reagent compositions [29]. A bicontinuous network is only obtained within a strict composition and condition regime. Otherwise, the result is isolated pore structures or powders.

### 2.3. Synthesis with template

Macroporous structures can be obtained by using ice, latex microspheres, polymeric foams and emulsion droplets as direct templates [30,31,32,33,34,35,36,37]. The procedure is quite simple: a hard template is suspended or immersed in a sol. After gelation (and drying), the template is removed (by calcination or washing) and results in a macroporous structure.

Furthermore, self-assembling surfactants and amphiphilic block copolymers can be used to prepare hierarchical macro-mesoporous oxides. However, it is challenging to obtain hierarchical pore structures in monolithic non-silica oxides and carbon. Nevertheless, some successful achievements are described later in this paper.

### 2.4. The Replica method

**Figure 3:**
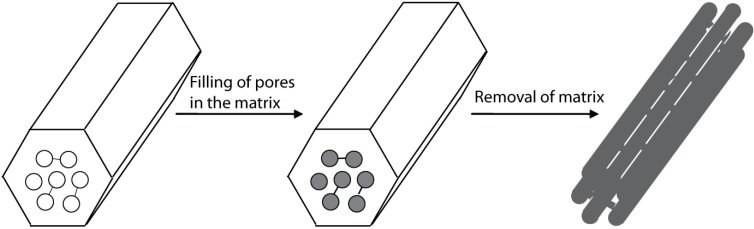
The replica method [39].

The replica method is an approach involving reproduction of the macro- and mesoporous structure of a template. Usually porous silica prepared by the sol-gel method is used as the rigid matrix template. One or more precursors are incorporated into the pores of the matrix by imbibition. After heat treatment, a continuous solid framework of the desired material is preserved within the pores. Subsequently, the matrix is removed by etching and both the macro- and mesostructures of the voids in the original matrix is replicated (see Figure 3) *i.e,.* the walls of the matrix have become the voids of the replica. [38,39,40,41]

A variety of materials have been obtained by this approach. In most cases it has been used to prepare porous carbon, noble metal, metal oxide and zeolitic ordered porous structures [42,43,44,45]. However, it is also possible to obtain nanowires [46] and mesoporous microspheres [47].

## 3. Monolithic Oxides

### 3.1. Alumina

Aluminium oxide is an important compound in many applications. Due to the thermal, chemical, and mechanical stability, alumina materials are often preferred over silica oxide materials. The possibility of preparing hierarchically porous monoliths will most likely broaden its application areas further.

**Figure 4:**
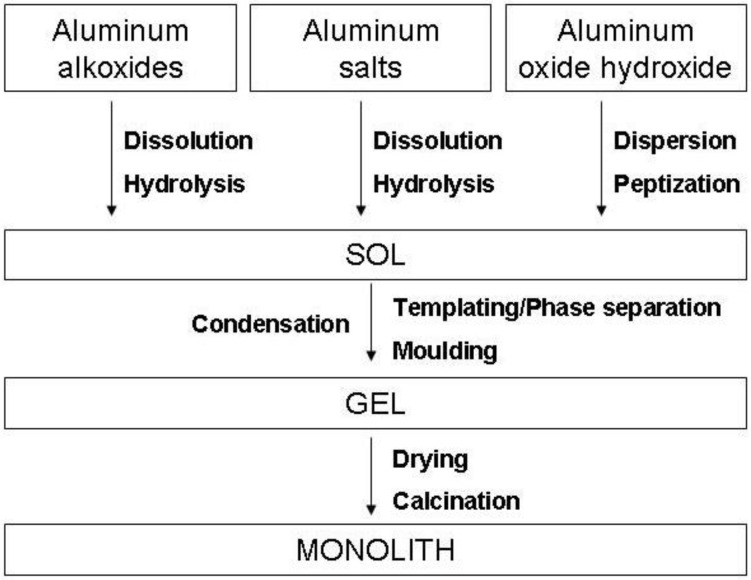
The Sol-gel alumina monolith preparation scheme.

Three classes of alumina precursors are typically used for sol preparation (1) aluminium alkoxides (2) inorganic alumina salts (3) aluminium oxide hydroxide (boehmite) or aluminium hydroxide (bayerite), either in the form of gel or as dispersed nanopowder (see Figure 4.).

The hydrolysis of aluminium alkoxides was first described by Yoldas in 1973 [48]. In this work it was shown that depending on the reaction temperature the hydrolysis and polycondensation can result in boehmite or amorphous aluminium monohydroxide. A preparation method for porous, transparent aluminium oxide films from aluminium alkoxide sols was also described [49]. This opened a new route within alumina sol-gel chemistry. Aluminium alkoxides are very reactive and the addition of chelating agents to control both the hydrolysis and condensation rate is often required [50,51,52]. Aluminium isopropoxide and aluminium sec-butoxide, were both used to obtain high-surface-area monolithic aerogels [53,54,55,56,57,58,59]. The first gamma alumina xerogel monoliths with hierarchical porosity and hexagonally ordered mesopores within macroporous walls were prepared by Li *et al.* [60]. In this approach aluminium isopropoxide was used as the sol-gel precursor, polyurethane foam as the macroporous template and triblock copolymer P123 as the mesoporous structure-directing agent.

It is well known that aluminium salts hydrolyze and condensate into aluminium oxide hydroxide gels under basic conditions [61,62]. However, aqueous aluminium salt solutions are acidic due to partial aluminium hydroxocomplex hydrolysis, as shown in equation 6 [63].

(6) $${\left[Al{\left(H_{2}O\right)}_{6}\right]}^{3+}+nH_{2}O={\left[Al{\left(OH\right)}_{n}{\left(H_{2}O\right)}_{6-n}\right]}^{(3-n)+}+nH_{3}O^{+}$$

Increasing the pH, shifts the equilibrium towards the right. During basification, Al^3+^ ions undergo several intermediates [19] and the subsequent condensation (via olation and oxolation) results in formation of polynuclear hydroxides or oxo-hydroxides, eventually leading to sol-gel transition.

Focus has been put on controlling the hydrolysis by gradual and homogenous increase of pH. A method utilizing propylene oxide as a gelation initiator was first described by Baumann *et al.* [64]. The epoxide acted as a proton scavenger during the sol-gel polymerization reaction, leading to a gradual increase of pH. The resulting monoliths were giving fragile, low-density (60-130 kg/m^3^), but high-surface-area (600-700 m^2^/g) aerogels. This approach has also been combined with the phase separation method, resulting in aerogels as well as more mechanically stable xerogels. Tokudome *et al.* [65,66] used poly(ethylene oxide) polymer as the porogen and propylene oxide as the proton scavenger in aqueous and ethanolic solutions of aluminium salts. The size and morphology of the macro- and mesopores of the monoliths were controlled by appropriate choices of the starting composition. Depending on the calcination temperature, the specific surface area of the as-synthesized materials varied from 182 to 512 m^2^/g. The same authors also showed that the thermal stability of the as-prepared materials (cylindrical monoliths with dimensions 5 × 5 × 5 mm) can be improved by doping the structure with lanthanum ions [67].

Both dry and gelatinous aluminium oxide hydroxide can be used to prepare stable sols. The gelation is then an effect of dewatering, concentration or introduction of acids or salts [68,69]. A preparation method of monolithic alumina based on microcrystalline boehmite was patented in 1976 by Kiovsky *et al.* [70]. Monoliths with high surface areas were made by controlled mixing of boehmite alumina aggregates and monobasic acids, such as formic or nitric acid. A free-flowing mass of boehmite aggregates was moulded into the desired shapes, dried and calcinated. The surface areas of the as-prepared material ranged from 10- 300 m^2^/g. The chemical homogenity of the microstructure was later improved by applying the sol-gel technique to this system [71]. Monoliths with tuned porosity can also be prepared from boehmite. Recently Zhao and Liu made hierarchically porous monoliths by imbibing polystyrene foam into alumina hydrosols prepared from pseudo-boehmite. [72]. After calcination at a high temperature the organic template was removed. The macropores were interconnected by meso and micropores and the hierarchical structure was controlled by surfactant addition and the calcination temperature.

### 3.2. Zirconia

Zirconia (ZrO_2_) can occur in three polymorphic crystal phases at atmospheric pressure: monoclinic (m), tetragonal (t), and cubic (c). m- ZrO_2_ is stable at low temperature and transforms into t- ZrO_2_ or c-ZrO_2_ at 1170 °C and 2370 °C, respectively [73,74]. Interesting optical properties [75], combined with thermal and chemical stability, make zirconia useful within areas such as: oral transplantology [76,77,78], solid oxide fuel cells [79], hydrogen production [80,81], biomass conversion [82], catalytic supports [83], chromatographic purification/separation of pharmaceuticals [84].

Hierarchical ZrO_2_ can be prepared both by standard templating methods [37,85] and without templating [86,87]. However, obtaining mechanically stable monolithic materials is a challenging task. As for alumina, zirconia monoliths can be prepared from alkoxy precursors. However, zirconium alkoxides have faster hydrolysis rates compared to aluminum or titanium alkoxides. This is due to larger positive partial charge of the zirconium atom, which enhances nucleofilic attacks on the zirconium atoms [88].

Konishi *et al.* [88] used zirconium n-propoxide as the precursor in a sol-gel transition accompanied by phase separation. The hydrolysis was carried out in an aqueous solution, while the gelation rate was controlled by the temperature and addition of nitric acid and N-methylformamide. Poly(ethyleneoxide) was used to induce phase separation. Both the phase separation and the gelation proceeded spontaneously in a mixed sol, and the macropore size was controlled in the range of 300 nm to 2 μm by varying the starting composition. Both the specific surface areas and mesopore sizes were influenced by the temperature of the hydrothermal treatment. Dimensions of the resulting cylindrical shape monoliths were ca. 20 × 5 × 5 mm. Another method for preparing zirconia monoliths was proposed by Randon *et al.* [8]. They utilized the phase separation method with acetic acid as the gelation inhibitor. The acetic acid formed stable complexes with the zirconia precursor which reduced the hydrolysis rate.

Recently, hierarchical zirconia monoliths were successfully synthesized using ZrCl_4_ [89] and ZrOCl_2_ [90,91] as precursors. ZrO(OH)_2_⋅xH_2_O gels were made by drop-wise addition of ammonia into cooled aqueous solutions of ZrOCl_2_⋅8H_2_O [90,91]. The crystalline zirconia phase depended on the calcination temperature. Monoliths can also be obtained by electrochemical methods. A method presented by Zhao et al. [92] was based on electrochemical hydrolysis, where pH was gradually increased by simultaneous oxidation of Cl^-^ anions and reduction of H^+^ cations according to following reactions:

(7) $$4ZrOCl_{2}+12H_{2}O⇄{\left[Zr_{4}{\left(OH\right)}_{8}{\left(H_{2}O\right)}_{16}Cl_{6}\right]}^{2+}+2Cl^{-}⇄{\left[Zr_{4}{\left(OH\right)}_{8}{\left(H_{2}O\right)}_{16}\right]}^{8+}+8Cl^{-}$$

(7) $$2{\left[Zr_{4}{\left(OH\right)}_{8}{\left(H_{2}O\right)}_{16}Cl_{6}\right]}^{8+}⇄{\left[Zr_{8}{\left(OH\right)}_{20}{\left(H_{2}O\right)}_{24}\right]}^{12+}+4H^{+}+4H_{2}O$$

The hydrolysis and condensation reactions are shifted towards the products, which may further condense and form inorganic networks at critical concentrations. Both critical concentrations and gelation time depend on the additives. It was reported that solvents with low dielectric constants decreased the stability of zirconia and promoted gelation. The drying method also influenced the material properties. The aerogel was a transparent with an average pore size of 9.7 nm and the surface area as high as 640 m^2^/g. The freeze dried gel, on the other hand, gave a microporous structure with surface area and mean pore size of about 400 m^2^/g and 0.6 nm, respectively. After calcination, the aerogel exhibited a mixture of m-ZrO_2_ and t-ZrO_2_, while the freeze dried gel had a single t-ZrO_2_ phase.

### 3.3. Titania

Titania (TiO_2_) has been applied within research areas such as photocatalysis [93], sensing [94] and separation science [95]. TiO_2_ exists in three mineralogical crystal phases: anatase, rutile and brookite. The anatase and brookite phases transform into the rutile phase at temperatures above 900 °C. The crystal phases have strong influence on the properties of titania. An example is that the rutile phase show lower photocatalytic activity than those for anatase and rutile anatase mixture, while the brookite phase is inactive [96,97].

Sol-gel methods for fabricating titania monoliths were proposed by Konishi *et al.* [98,99,100] and Fujita *et al.* [17]. The methods were based on phase separation and the starting sols were either titania powder or prepared from titanium n-propoxide precursors. In one method [99], the starting hydrosol consists of anatase nanocrystals, nitric acid, formamide and poly(ethylene oxide). Due to the electrostatic repulsion of positively charged titania nanoparticles in strongly acidic conditions (the isoelectric point of titania is at pH = 5.5 – 6.0), the powder was well dispersed in the solution. The pH of the sol was gradually increased during decomposition of formamide to ammonia. Gelation was induced as a result of aggregation of particles. Simultaneously, phase separation driven by the reduction of poly(ethylene oxide) miscibility took place. The phase-separation process and the interporous connectivity were enhanced by increasing the molecular weight of the polymer. The size and volume of the pores could be controlled by adjusting the polymer and titania concentrations and materials with surface areas as high as 350 m^2^/g and macropore volumes of about 0.34 cm^3^/g were prepared. Unfortunately, the monoliths were very fragile due to weak interactions between the aggregated colloidal particles. The problem of mechanical instability was dealt with by developing a method based on titanium n-propoxide [98]. Titanium alkoxides are highly reactive, but optimization of the reaction mixture and drying procedure made it possible to produce relatively hard and stable cylindrical monoliths, a few centimeters long with diameter of *circa* 4 mm. The fracture stress (stress which cause visible cracks) measured for these monoliths was 4000 MPa [100]. The resulting monoliths calcinated at 300 °C, exhibited surface areas of 150 m^2^/g and a pore volume of about 0.46 cm^3^/g.

Monoliths fabricated from titanium i-propoxide have also been achieved by using glycerol to slow down the hydrolysis and condensation rates [101]. The hydrolysis rate of the precursor was also influenced by the amount of water in the reaction mixture. As in all phase separation systems, poly(ethylene oxide) concentration and average molecular weight mostly influenced the morphology of the resulting monoliths. Macroporous materials with bicontinous structure and grainy morphology was obtained by varying the poly(ethylene oxide) concentration. Titania monoliths with interconnected pore networks appeared at polymer concentrations, where spinodal decomposition occurred. Monoliths calcinated at 600 °C had a maximum surface area of 21.5 m^2^/g (BET surface area) and a pore volume of 0.8 cm^3^/g (calculated from mercury intrusion).

A templating method using xantan carbohydrate polymer as the template was used by Shchipunov and Postnova [102] to synthesize titania monoliths. The resulting areogel had pore diameters between 100 and 250 nm. The morphology of the material depended on the amount of water and precursor (titanium (IV) isopropoxide) in the synthesis. The grain size could be decreased by increase of water concentration. The variation of the precursor content leads to fibrillar, granular or plate-like morphologies.

### 3.4. Other materials

A brief description of preparation methods for porous monoliths of other oxides, sulfides, carbon and metals is given in this section. The most useful methods were the replica method [45] and direct gelation, rather than the phase separation or templating methods. See Table 1.

**Table 1:** Overview of monolithic mesoporous materials synthesized using different approaches.

Material	Preparation Method
LiFePO_4_/Carbon Composite [11]	replica
CdSe/ZnS [12]	direct gelation
Ag_2_Se [13]	direct gelation
CdS-Ag [103]	direct gelation
MnO_2_, Mn_2_O_3_, SnO_2_, Co_3_O_4_ [42,104]	replica
MgO [105]	direct gelation
Fe_2_O_3_ [106]	direct gelation
FeOOH [107]	direct gelation
NiO-ZnO [108]	direct gelation
SnO_2_ [20]	direct gelation
ZnO [109]	direct gelation
Silver [110]	template synthesis
Carbon [40,111,112,113]	replica
SiC [114]	replica
MgAl_2_O_4_ [115]	replica

Amorphous magnesium oxide monoliths [105] were made by hydrolysis and condensation of Mg-alkoxide in the presence of glycerol and acetic acid. The acetic acid initialized Mg-O, Mg-H and Mg-OH bonds formation, whereas glycerol slowed down the gelation, resulting in crack-free gels. The materials did not exhibit any intermediate crystalline products, such as brucit or Mg acetate. Quantum-dot xerogels and aerogels can be made by the gelation of CdSe/ZnS sols [12]. A similar method with subsequent ion exchange Zn by Ag resulted in Ag_2_Se monoliths [13]. A LiFePO_4_/carbon composite [11] was synthesized by imbibing a lithium iron phosphate precursor solution into a preformed hierarchically porous carbon monolith. The carbon monolith was synthesized by the replica method from a silica monolith [116,117]. Carbon monoliths were also utilized as the matrix during preparation of MgAl_2_O_4_ [115]. A zinc oxide aerogel monolith was prepared by the hydrolysis of zinc nitrate using propylene oxide as proton scavenger. The material had flower-like morphology and photoluminescence properties [105]. Also Fe_2_O_3_ and FeOOH monoliths were prepared according to this approach, starting from ethanolic solutions of FeCl_3_ or Fe(NO_3_)_3_ [106,107]. The ionic liquid N-(2-hydroxyethyl) ammonium formate was simultaneously used as the solvent, template and reducing agent in a microwave synthesis of silver monoliths with silver nitrate as the metal precursor. The resulting porous metal had macropores with a diameter of 600 nm [110]. Bellayer *et al.* also utilized ionic liquids as the solvent in the sol-gel synthesis of SnO_2_ monoliths [20]. Smått *et al.* [42,104] applied the replica method to prepare manganium, cobalt and tin oxide cylindrical shape monoliths (dimensions *circa* 10 × 5 × 5 mm) by imbibing monolithic silica matrices with a saturated solution of the relevant inorganic salts. After drying, the silica matrices were removed by etching with NaOH or HF solutions. The resulting monoliths had surface areas from 30 to 70 m^2^/g and pore volumes from 0.075 to 0.119 cm^3^/g. Carbon monoliths were also obtained by replication of silica monoliths. Matrices were impregnated using a carbon precursor (e.g. furfuryl alcohol), and subsequently heat treated in order to polymerize and carbonize the precursor. Finally, the silica matrix was removed by etching [40].

## 4. Conclusions

Recent developments in sol-gel methods have afforded the preparation of a variety of porous inorganic monoliths. Utilization of the phase separation and replica methods makes it possible to fabricate monoliths using materials that otherwise would not result in monoliths. The phase-separation technique has helped to overcome the problem of cracking during the drying procedure by introducing interconnected macroporosity without the need of a template. Difficulties, such as high reactivity of precursors, were solved by the introduction of gelation inhibitors. Thus, simultaneous gelation and phase separation could be obtained. A great advantage of this method is the resulting bicontinous network of channels with a homogenous pore distribution in the whole material. Casting different kinds of materials into matrices with hierarchical pore structures afforded the preparation of monoliths with the same pore structure using different materials.
